# Underestimation of Prostate Cancer Grade in Transperineal Fusion Biopsy and Its Predictive Factors: Correlation of Biopsy Findings with Post-Da Vinci Radical Prostatectomy Specimens

**DOI:** 10.3390/jcm15072780

**Published:** 2026-04-07

**Authors:** Hubert Andrzej Krzepkowski, Tomasz Ząbkowski, Maciej Walędziak, Tomasz Waldemar Kamiński, Hubert Dąbrowski, Tomasz Syryło

**Affiliations:** 1 Department of General, Oncological and Functional Urology, Military Institute of Medicine—National Research Institute, 04-141 Warsaw, Poland; 2 Department of General, Oncological, Metabolic and Thoracic Surgery, Military Institute of Medicine—National Research Institute, Szaserów 128 St., 04-141 Warsaw, Poland; 3 Faculty of Medicine, University of Warsaw, 04-141 Warsaw, Poland; 4 VERSITI Blood Research Institute, 8727 W Watertown Plank Road, Wauwatosa, WI 53226, USA; 5 Psychiatric Ward of the Mental Health Center in Kielce, 59 Kosucińskiego, 25-450 Kielce, Poland; 6 Świętokrzystkie Center of Psychiatry in Morawica, 5 Spacerowa, 26-026 Morawica, Poland

**Keywords:** prostate cancer, radical prostatectomy, Gleason score, Gleason score upgrading, histopathology, risk stratification, prostate-specific antigen, biopsy accuracy, prostate volume assessed by magnetic resonance imaging, PSA density

## Abstract

**Background/Objectives**: An accurate preoperative assessment of prostate cancer malignancy is crucial for risk stratification and selection of the optimal treatment strategy. This study assessed the concordance of Gleason scores between MRI–TRUS fusion biopsy and radical prostatectomy specimens, and identified clinical and histopathological factors associated with post-procedural Gleason score upgrading. **Methods**: This retrospective analysis involved patients who underwent transperineal MRI–TRUS fusion biopsy followed by radical prostatectomy from 2020 to 2025. Concordance, upgrading, and downgrading of the Gleason score were assessed by comparing biopsy results with the final histopathological examination. Clinical parameters (age, PSA level, prostate volume, and PSA density) and histopathological features of biopsies (Gleason score and percentage of prostate lobes affected by cancer) were analyzed. Multivariate logistic regression models were stratified by PSA level (<10 ng/mL and >10 ng/mL). **Results**: Gleason score concordance was found in 53.1% of the 603 patients analyzed, upgrading in 29.9%, and downgrading in 17.1%. Higher Gleason scores on biopsy were independently associated with a lower risk of upgrading in the entire cohort and in both PSA subgroups. Larger tumor extent on biopsy was associated with a lower risk of upgrading, with heterogeneous dependencies between prostate lobes. The other clinical parameters showed no independent association with upgrading. **Conclusions**: Gleason score upgrading remains common after radical prostatectomy. The risk of this progression is primarily related to the histopathological features of the biopsy rather than to baseline clinical parameters, reflecting the limitations of biopsy as a sampling method and the biological heterogeneity of prostate cancer.

## 1. Introduction

Prostate cancer (PCa) is one of the most common malignant tumors among males worldwide and remains a serious clinical and epidemiological problem. The number of new diagnoses is constantly growing, which is primarily due to the aging population, the widespread use of PSA testing, and increasingly advanced diagnostic imaging methods. According to GLOBOCAN data for 2022, PCa was the second most common cancer diagnosed in men and was also one of the leading causes of cancer deaths, ranking fifth globally [1]. European countries, which are among the regions with the highest incidence rates in the world, show significant differences across individual countries. Despite these regional differences, the overall epidemiological picture remains similar: the number of new diagnoses remains high whereas mortality is gradually declining, which may be attributed to the advancement of diagnostic methods and increasingly effective treatment options [2]. PCa takes many different biological forms, ranging from slow-growing lesions confined to the gland to highly aggressive forms with a high tendency to metastasize. For this reason, a key step in the management process is to accurately determine individual disease risk, based primarily on a detailed and precise histopathological evaluation [3]. The prostate biopsy remains the main method for making a preliminary diagnosis. Although this technique is widely used, it still carries a risk of diagnostic error. This is due, among other factors, to the limited amount of material collected, significant variability within the tumor, and uneven distribution of neoplastic foci [4]. One of the significant challenges in modern urology continues to be the underestimation of tumor malignancy in biopsies. This refers to a situation where the Gleason score or Grade Group derived from biopsies is either lower or higher than that found in the sample after radical prostatectomy. Such discrepancies have significant clinical implications and may lead to incorrect risk profiling, which in turn may result in an inappropriate treatment strategy that is either too lenient an approach within active surveillance or a delay in implementing radical treatment in patients whose cancer is more advanced [5,6]. Numerous analyses comparing biopsy results with histopathological evaluation after radical prostatectomy have shown that a significant proportion of patients, from approximately 30% to over 70%, experience a change in tumor grade (degree of tumor malignancy). Upgrading is the most observed finding, although downgrading also occurs in some patients. This phenomenon clearly indicates the limitations of biopsy as a standalone tool for accurate assessment of oncological risk [6,7]. Recent research confirms the fundamental importance of accurate grading of PCa. According to data presented by Epstein et al., the Gleason system, modified in 2014, remains the most important and reliable prognostic factor in PCa. The authors emphasize that precise grading not only allows for an accurate assessment of tumor aggressiveness but also better predicts the risk of recurrence, clinical course, and overall survival, making it a key element in therapeutic decision-making [8]. Recent scientific reports have emphasized that a number of clinical and pathomorphological parameters influence the risk of underestimating the stage of cancer in a biopsy. The most important of these include PSA density, total prostate volume, and the location of the neoplastic lesion. Attention has also been drawn to the presence of features indicative of a more aggressive course of the disease, such as vascular invasion, neural involvement, invasion beyond the prostate capsule, or the presence of cancer in the seminal vesicles. The analysis of these elements in correlation with the histopathological result after prostatectomy is an important area of research, which allows for a more precise determination of risk and tailoring of the therapeutic strategy to individual patients [9,10,11]. At the same time, advances in diagnostic techniques such as multiparametric magnetic resonance imaging (mpMRI) and MRI-targeted biopsies have significantly reduced the risk of obtaining material that does not reflect the actual nature of the tumor. However, even fusion biopsy does not eliminate the risk of misdiagnosis, especially in patients with multifocal tumors or tumors that are poorly visible on MRI [12]. Considering the difficulties described above, the objective of this study was to assess the concordance rate between the grade of PCa determined in a biopsy and the histopathological results after radical prostatectomy. The analysis covered selected clinical parameters, such as PSA level, PSA density, and prostate volume, as well as the location of the lesion, its stage, and the presence of features indicating a more aggressive nature. The results obtained may serve as a basis for improving the diagnostic process and more accurately selecting patients for optimal treatment strategies.

## 2. Materials and Methods

This retrospective analysis involved patients who underwent radical prostatectomy at the Department of General, Oncological, and Functional Urology of the Military Medical Institute–National Research Institute in Warsaw. Overall, the study included 603 eligible patients aged 39–86 years who were scheduled for radical prostatectomy. The patients underwent transperineal fusion biopsy with a Gleason score of ≥6 and prostate MRI. MRI parameters were not included in this analysis. Data were collected from the hospital’s electronic medical records system after patient hospitalization. The study included individuals who underwent prostatectomy between 2020 and 2025. The comprehensive data analysis included a wide range of clinical and demographic parameters, including patient age, PSA level, prostate volume assessed by magnetic resonance imaging, PSA density, DRE, tumor location in the prostate, and degree of prostate involvement by the tumor. PSA levels in patients receiving 5-alpha-reductase inhibitors were adjusted by doubling the measured PSA value, in accordance with established clinical practice. Concordance was defined as an identical Gleason score (ISUP grade grouping) obtained in the biopsy specimen and in the final histopathological examination after radical prostatectomy. Upgrading was defined as any increase in the Gleason score or ISUP grade grouping in the postoperative specimen compared to the biopsy result, whereas downgrading was defined as any decrease in the Gleason score or ISUP grade grouping in the postoperative specimen compared to biopsy evaluation. In procedures performed using the da Vinci robotic system, the transperitoneal approach was used as the primary method of access. Patients who underwent robot-assisted radical prostatectomy (RARP) were eligible for the procedure according to the National Health Fund criteria. The study included patients diagnosed with PCa with a Gleason score of 6–10 (ISUP grades 1–5), patients with organ-confined disease (clinical stage cT1–T2 N0 M0) or locally advanced disease (cT3a–b N0–1 M0), without evidence of distant metastases (M0), as confirmed by negative bone scintigraphy or whole-body magnetic resonance imaging. In addition, all included patients showed preserved erectile function, defined as an IIEF-5 score > 21. Exclusion criteria included lack of patient consent, exclusion due to anesthetic risk, and the presence of metastatic cancer.

Statistical analyses were performed using standard statistical software. Continuous variables were expressed as mean ± standard error of the mean (SEM) or median with interquartile range (IQR), as appropriate, while categorical variables were presented as counts and percentages. Comparisons between groups (downgrading, concordance, upgrading) were performed using one-way analysis of variance (ANOVA) for continuous variables and appropriate post hoc tests for pairwise comparisons. Categorical variables were compared using the chi-square test. Univariate analyses were initially conducted to identify variables associated with Gleason score upgrading. Variables with potential clinical relevance were subsequently included in multivariate logistic regression models to identify independent predictors of upgrading. Results were reported as regression coefficients with standard errors and 95% confidence intervals. Additional multivariate logistic regression analyses were performed in subgroups stratified by PSA level (<10 ng/mL and ≥10 ng/mL). Correlation between clinicopathological variables was assessed using Spearman’s rank correlation coefficient. Multicollinearity between variables was assessed using correlation analysis. No strong correlations between independent variables were observed, indicating a low risk of multicollinearity. Formal goodness-of-fit statistics were not calculated due to the retrospective nature of the dataset; therefore, model performance should be interpreted with caution. All statistical tests were two-sided, and a *p*-value < 0.05 was considered statistically significant. All the statistical analysis has been done using GraphPad Prism (La Jolla, CA, USA).

## 3. Results

The analysis included 603 patients who underwent radical prostatectomy. The mean age of the patients was 67.7 ± 0.28 years. The median concentration of PSA in the entire cohort was 7.0 ng/mL (25th to 75th percentile: 5.15 to 10.0). The average prostate volume was 46.1 ± 0.97 mL. The average duration of the surgical procedure was 165 ± 2.11 min, whereas the mean surgical margin width was 4.48 ± 0.65 mm.

Based on the concordance of the Gleason classification between biopsy and postoperative samples, patients were divided into three groups: downgrading (*n* = 103), concordance (*n* = 320), and upgrading (*n* = 180). No statistically significant differences were found between the groups in terms of patient age, baseline PSA values, digital rectal examination results, duration of surgery, or surgical margin width (all *p* > 0.05).

Differences were observed between groups in prostate volume: the average prostate volume was 47.2 ± 2.66 mL in the downgrading group, 44.2 ± 1.19 mL in the concordance group, and 49.1 ± 1.95 mL in the upgrading group. The univariate analysis revealed significant differences between the groups, whereas the direct comparison analysis showed that the difference between the concordance and upgrading groups did not reach statistical significance (*p* = 0.0805) but showed a statistical trend toward higher prostate volume in the upgrading group.

Clinical and perioperative characteristics of patients are presented in Table 1. In the analyzed cohort, Gleason score concordance between biopsy and post-prostatectomy specimens was found in 320 patients (53.1%), upgrading in 180 patients (29.9%), and downgrading in 103 patients (17.1%). In the upgraded group, a significant increase in the Gleason score between the biopsy and postoperative assessment was observed (*p* < 0.0001), whereas in the downgraded group, a significant decrease in this score was observed (*p* < 0.0001); in the concordance group, no significant differences between the assessments were found (*p* = ns).

**Figure 1:**
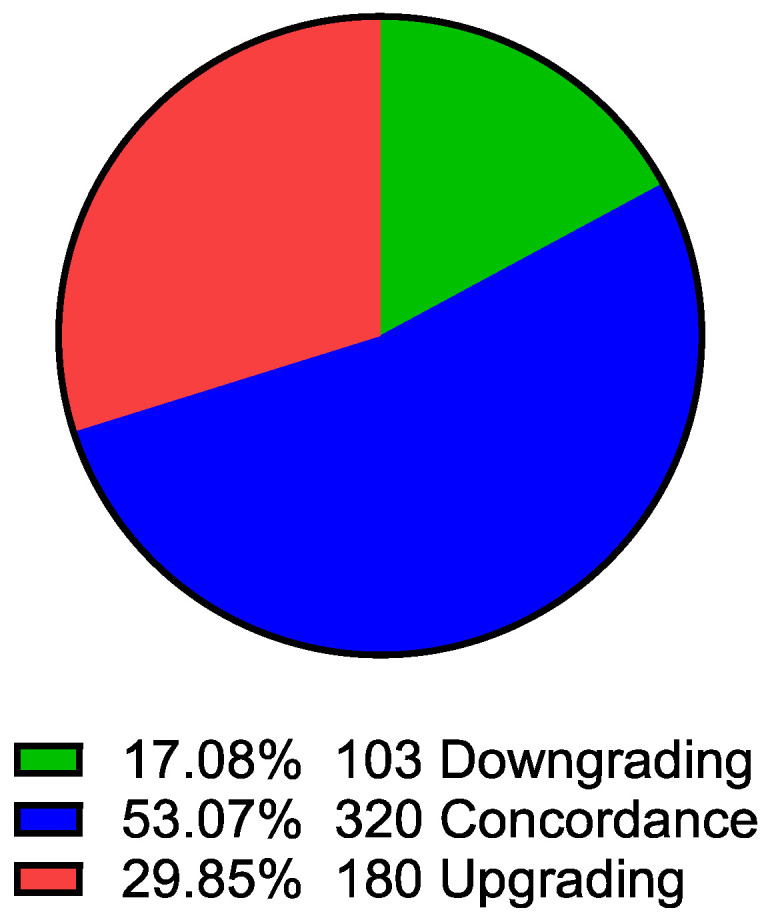
Distribution of Gleason score changes between biopsy and radical prostatectomy specimens.

Figure 1 illustrates the proportion of patients exhibiting downgrading (green), concordance (blue), and upgrading (red) of Gleason score following radical prostatectomy compared with initial MRI–TRUS fusion biopsy findings. Concordance was observed in 320 patients (53.1%), upgrading in 180 patients (29.9%), and downgrading in 103 patients (17.1%) within the analyzed cohort (*n* = 603).

**Figure 2:**
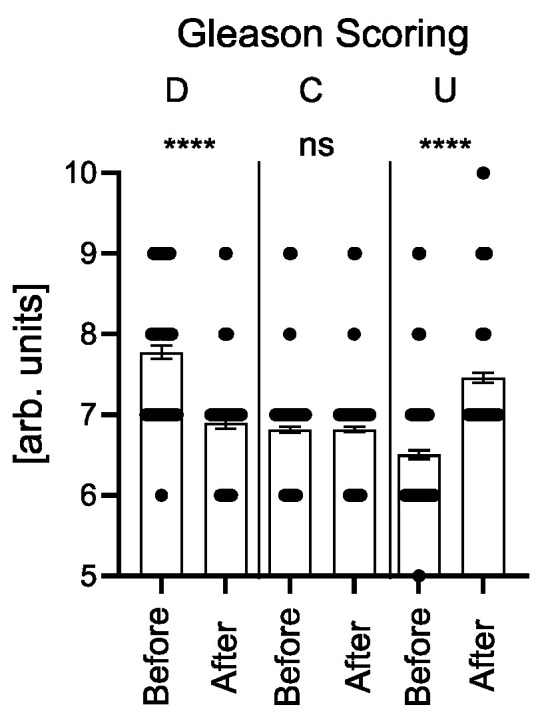
Comparison of Gleason scores before biopsy and after prostatectomy in the downgrading, concordance, and upgrading groups. Asterisks denote levels of statistical significance (**** *p* < 0.0001), whereas “ns” indicates a non-significant difference.

Figure 2 shows the comparison of Gleason scores before biopsy and after prostatectomy in the downgrading, concordance, and upgrading groups.

Table 2 presents the results of the multivariate logistic regression analysis assessing independent factors associated with the risk of Gleason score upgrading after prostatectomy. In addition to basic clinical variables, the model included parameters that describe tumor characteristics and detectability estimates, including the Gleason score on biopsy and the percentage of individual prostate lobes affected by the neoplastic lesion. In the multivariate analysis, a higher Gleason score on biopsy was significantly associated with a lower risk of upgrading after prostatectomy. A larger extent of neoplastic change, expressed as a percentage of prostate lobe involvement, also demonstrated a significant association with a reduced risk of upgrading. This correlation was observed for the left prostate lobe, whereas the corresponding parameter for the right lobe did not reach statistical significance. The other variables included in the model, including patient age, PSA level, prostate volume, and duration of surgery, showed no independent association with Gleason score upgrading after adjustment for covariates. The magnitude and direction of the regression coefficients indicate that higher Gleason scores on biopsy were associated with a substantially reduced likelihood of upgrading, whereas the effects of clinical variables such as PSA and prostate volume were small and not statistically significant. The inclusion of additional parameters describing tumor characteristics did not change the direction or significance of the associations observed for the main predictors of upgrading in the multivariate analysis.

Table 3 presents the results of the multivariate logistic regression analysis performed in a subgroup of patients with baseline PSA > 10 ng/mL. The model included the PSA level, prostate volume, Gleason score in biopsy, and the percentage of right and left prostate lobe involvement by cancer. In this analysis, PSA levels did not show a significant independent association with the risk of Gleason score upgrading after prostatectomy. Prostate volume was also not significantly associated with upgrading after adjustment for other variables. The location of the tumor in individual lobes of the prostate, assessed as the presence of a tumor and its percentage, did not show independent predictive ability for the risk of upgrading in this subgroup of patients. A higher Gleason score in the biopsy remained significantly associated with a lower risk of upgrading after prostatectomy, even when the analysis was limited to patients with PSA > 10 ng/mL. Furthermore, a higher percentage of cancer in the right lobe of the prostate was associated with a reduced risk of upgrading, whereas the corresponding parameter for the left lobe did not reach statistical significance. The other variables considered did not show a significant independent association with Gleason score upgrading after adjusting for covariates in the model.

Table 4 presents the results of the multivariate logistic regression analysis performed in a subgroup of patients with baseline PSA < 10 ng/mL. The model included PSA level, prostate volume, Gleason score in biopsy, and the percentage of right and left prostate lobe involvement by the neoplastic lesion. In this analysis, PSA levels did not show a significant independent association with the risk of a Gleason score upgrading after prostatectomy. The volume of the prostate gland and the location of the tumor in individual lobes of the prostate, assessed as the percentage of the lobe involved by the tumor, were also not significantly associated with the risk of upgrading after adjusting for other variables included in the model. The only factor showing a statistically significant independent association with the risk of upgrading in this subgroup was the Gleason score on biopsy. A higher Gleason score in biopsy material was associated with a lower risk of Gleason grade upgrading after prostatectomy. The other variables did not reach statistical significance in the multivariate analysis.

In the analysis of Gleason grade upgrading frequency stratified according to the PSA level, upgrading occurred in 28.3% of patients in the group with PSA < 10 ng/mL (125/441) and in 35.6% of patients in the group with PSA > 10 ng/mL (53/149). This difference did not reach statistical significance (*p* = 0.12). Despite lower baseline PSA levels, upgrading remained common in the subgroup of patients with PSA < 10 ng/mL.

**Figure 3:**
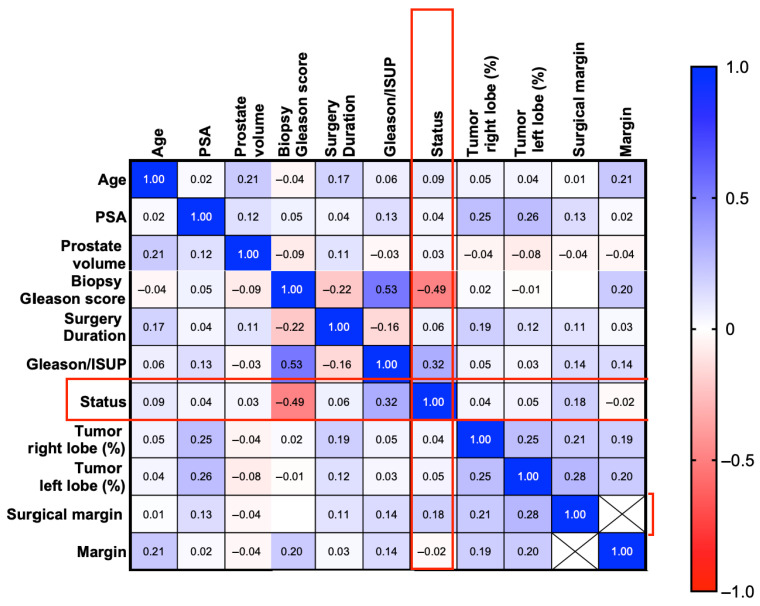
Correlation matrix of clinicopathological and perioperative variables. Colors represent the strength and direction of Spearman correlation coefficients, ranging from negative (blue) to positive (red) values. The numerical values within the cells indicate correlation coefficients (r). Red frames highlight correlations involving the “status” variable (downgrading, concordance, and upgrading).

As shown in Figure 3, a moderate positive correlation was found between the Gleason score in the biopsy and the postoperative Gleason/ISUP classification (r = 0.53), whereas a moderate negative correlation was observed between the Gleason score in the biopsy and the concordance status (downgrading/concordance/upgrading; r = −0.49) indicating only limited predictive strength of this variable when considered in isolation. The status also showed a positive correlation with the postoperative Gleason/ISUP classification (r = 0.32). Other correlations between status and clinical parameters, such as age, PSA, and prostate volume, had low correlation coefficients. The percentage of involvement of the right and left prostate lobes by the tumor showed a moderate positive correlation with each other (r = 0.25) and weak correlations with surgical margin and clinical status. The prostate volume correlated weakly positively with the age of the patient (r = 0.21), while the PSA level showed only weak correlations with the other variables analyzed. Strong correlations between most clinical parameters and the upgrading status were not observed, confirming the lack of significant collinearity between the variables included in the further multivariate analyses.

**Figure 4:**
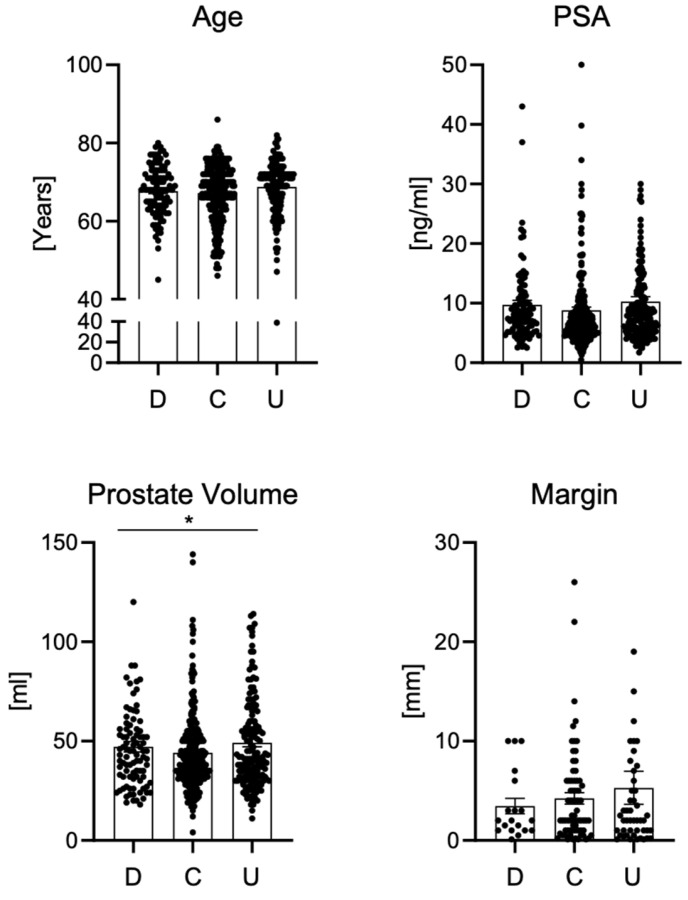
Comparison of age, PSA level, prostate volume, and surgical margin among patients with downgrading, concordance, and upgrading. An asterisk (*) indicates a statistically significant difference (*p* < 0.05).

Figure 4 shows a comparison of selected clinical and pathological parameters between groups of patients with downgrading, concordance, and upgrading of the Gleason score after prostatectomy. No significant differences in patient age were found between the analyzed groups. The age distribution was comparable in all three categories, and the mean and median values remained at a similar level. Furthermore, baseline PSA levels did not differ significantly between the downgrading, concordance, and upgrading groups. A wide range of PSA values was observed in all groups, but there were no significant differences between the medians or distributions of variability. In contrast to the above parameters, the prostate volume showed significant differences between the groups. Patients in the upgrading group presented larger prostate volumes compared with those in the concordance group. However, this difference did not reach statistical significance (*p* = 0.0805) and should be interpreted as a statistical trend. In the downgraded group, prostate volume ranged within intermediate values. Analysis of surgical margin width did not reveal significant differences between groups. The distribution of margin values was similar in the downgrading, concordance, and upgrading groups, and the observed differences were not statistically significant.

## 4. Discussion

This study assessed the concordance of the Gleason score between prostate biopsy performed using fusion MRI-TRUS and the final histopathological results following radical prostatectomy. The results obtained indicate that Gleason score upgrading remains a common clinical phenomenon, occurring in a significant percentage of patients despite the use of MRI-guided targeted biopsy [13,14]. These observations confirm that even advanced diagnostic strategies do not eliminate the risk of underestimating the biological aggressiveness of PCa prior to surgical treatment. The frequency of upgrading of the Gleason score observed in the analyzed cohort is within the range of values described in previous studies, in which discrepancies between biopsy and postoperative assessment were found in a significant percentage of patients [15,16]. The scale of this phenomenon depends on the biopsy technique used, the characteristics of the population studied, and the criteria for eligibility for radical cancer treatment. These results provide data relating to the population of patients diagnosed using MRI–TRUS fusion biopsy, allowing for their relevance to contemporary clinical practice [17,18,19]. The analysis of basic clinical parameters showed the limited predictive value of variables such as PSA level and prostate volume in predicting Gleason score upgrading. These associations did not persist after adjustment for covariates or in analyses stratified by PSA level. These results are consistent with observations that classic clinical markers, despite their usefulness in diagnosis and disease monitoring, do not always reflect the actual biological heterogeneity of PCa [20,21].

In contrast to clinical parameters, the histopathological features of the biopsy material proved to be the most stable predictors of concordance in malignancy assessment. A higher Gleason score in the biopsy was consistently associated with a lower risk of upgrading after prostatectomy, in both the analysis of the entire cohort and in the subgroup analyses. Similarly, a larger extent of neoplastic change was associated with a lower risk of underestimating malignancy. These observations underscore the importance of representative histopathological sampling, even in cases where biopsy is performed using MRI. It should be noted that the persistence of upgrading despite the use of fusion biopsy indicates the existence of limitations related not only to the location of tumor foci, but also to their internal heterogeneity. Regardless of the technique used, biopsy remains a sampling method limited to selected tumor fragments, which may lead to the omission of areas with higher malignancy. This issue has been the subject of numerous analyses in the literature and is an aspect that should be taken into consideration when interpreting the results obtained [22,23]. An interesting feature of this study was the observed asymmetry between the prostate lobes in terms of the relationship between tumor extent and the risk of upgrading. These differences were not uniform and were not present in all analyses, which limits the possibility of their unambiguous interpretation. It should be emphasized that the spatial characteristics of neoplastic lesions in the prostate are complex and can be modified by many factors, including the biopsy technique, the distribution of neoplastic foci, and individual anatomical differences.

The analyses stratified by baseline PSA level showed that classic clinical parameters have limited independent predictive value in assessing the risk of Gleason grade upgrading. In both the PSA > 10 ng/mL and <10 ng/mL groups, the absolute PSA value was not significantly associated with the risk of upgrading after adjusting for covariates, thus confirming its limited usefulness as a standalone predictor of histological malignancy. In both subgroups, the Gleason score in the biopsy remained a significant predictive factor, with a higher degree of tumor differentiation associated with a lower risk of upgrading after prostatectomy. In the subgroup of patients with PSA > 10 ng/mL, prostate volume and spatial parameters describing the location and percentage of prostate lobes affected by the neoplastic lesion did not show significant independent predictive value. Only a higher percentage of cancer in the right lobe of the prostate was associated with a reduced risk of upgrading, with no corresponding relationship on the left side. In the subgroup of patients with PSA < 10 ng/mL, the only independent predictor of upgrading remained the Gleason score in the biopsy, whereas the other clinical and spatial variables did not reach statistical significance. Despite lower baseline PSA levels, upgrading was common in this subgroup of patients, and the upgrading rate did not differ significantly between the PSA < 10 and PSA > 10 ng/mL groups. The results obtained indicate heterogeneity in the mechanisms leading to upgrading depending on the baseline clinical characteristics of patients, with histopathological features of biopsies being of key importance in both subgroups. Similar observations have been repeatedly reported by other authors, who indicate that after considering histopathological biopsy variables, the absolute PSA value loses its independent predictive value for Gleason score upgrading. In the multivariate analysis, PSA was often superseded by features that directly reflected the histological differentiation of the tumor, whereas its role remained limited to that of a marker of overall disease burden [24,25].

In the present study, only transperineal MRI–TRUS fusion biopsy was used prior to radical prostatectomy, reflecting the diagnostic protocol implemented at our institution during the study period. Although transrectal biopsy remains widely used in clinical practice, the findings of this study should primarily be interpreted in the context of the transperineal biopsy approach.

A major limitation of this study is the lack of inclusion of detailed MRI-derived parameters, such as PI-RADS score, lesion size, or lesion location, as independent variables in the predictive models. Although MRI–TRUS fusion biopsy was used, MRI served primarily as a guidance tool rather than as a source of quantitative imaging data. The absence of these variables may have limited the predictive performance of the models, as imaging features are known to provide additional information on tumor localization, visibility, and biological heterogeneity. This approach allowed us to focus on the relationship between the fusion biopsy results and the postoperative specimen, without the additional influence of imaging variables. This study has several additional limitations. First, it was conducted at a single center, which may limit the generalizability of the findings to broader patient populations. Second, the retrospective design introduces a risk of selection bias, as only patients who underwent both biopsy and radical prostatectomy were included. Third, the predictive models were not externally validated, which limits their immediate clinical applicability and requires confirmation in independent cohorts.

Analysis of the concordance between biopsy results and the final histopathological diagnosis after prostatectomy indicated that, in addition to upgrading, downgrading and cases of complete Gleason score concordance also played an important role. These observations highlight the bidirectional nature of diagnostic discrepancies, resulting from the limitations of biopsy as a sampling method and from the histological heterogeneity of PCa. The results of this study indicate that concordance between biopsy and postoperative specimens occurs only in some patients, which confirms the limited ability of biopsy findings to fully reflect the actual biological aggressiveness of the tumor. The phenomenon of downgrading may indicate an overestimation of the degree of malignancy in the biopsy material, potentially associated with preferential sampling of fragments with a higher degree of differentiation or with an overrepresentation of a more aggressive tumor component in the limited diagnostic material [26].

The simultaneous presence of Gleason score upgrading, downgrading, and concordance emphasizes that biopsy results should be interpreted as an approximate rather than a definitive assessment of PCa malignancy. The data obtained reinforce the need for cautious interpretation of the preoperative Gleason grading, especially in the context of therapeutic decision-making, and indicate the need to consider the risk of histopathological discordance regardless of the initial clinical characteristics of patients [27].

## 5. Conclusions

In this study, Gleason score upgrading occurred in 29.9% of patients with PCa, with concordance observed in 53.1%, and downgrading in 17.1%. A higher Gleason score on biopsy was independently associated with a lower risk of upgrading in the analysis of the entire cohort and in subgroups of patients with PSA < 10 ng/mL and >10 ng/mL. Greater tumor extent, expressed as the percentage of prostate lobe involvement, was associated with a reduced risk of upgrading, although this relationship was not uniform for both lobes. PSA level, prostate volume, and patient age showed no independent association with the risk of upgrading in multivariate analyses. The rate of upgrading did not differ significantly between patients with PSA < 10 ng/mL and >10 ng/mL. Correlation analysis revealed moderate concordance between the Gleason score in the biopsy and the postoperative grading, and there were no strong associations between upgrading and classic clinical parameters.

## Figures and Tables

**Table 1:** Comparison of clinical and perioperative characteristics of patients with downgrading, concordance, and upgrading. * *p* < 0.05.

Variable	(D)owngrading (*n* = 103)	(C)oncordance (*n* = 320)	(U)pgrading (*n* = 180)	ANOVA	Head-to-Head Comparison
Age [years]	67.7 ± 0.65	67.1 ± 0.39	68.8 ± 0.51	ns	ns
PSA [ng/mL]	9.72 ± 0.75	8.8 ± 0.54	10.3 ± 0.86	ns	ns
Prostate volume [mL]	47.2 ± 2.66	44.2 ± 1.19	49.1 ± 1.95	*	C vs. U, *p* = 0.0805
DRE	0.56 ± 0.56	0.51 ± 0.03	0.53 ± 0.04	ns	ns
Surgery duration [minutes]	158 ± 5.57	167 ± 2.81	165 ± 3.81	ns	ns
Margin [mm]	3.45 ± 0.78	4.24 ± 0.57	5.3 ± 1.66	ns	ns

**Table 2:** Logistic regression parameter estimates for predictors of upgrading/downgrading, including age, PSA, prostate volume, biopsy Gleason score, and tumor involvement of each lobe.

Parameter Estimates	Variable	Estimate	Standard Error	95% CI (Profile Likelihood)	*p* Value
β0	Intercept	0.6513	2.793	−4.828 to 6.194	
β1	Age	0.06303	0.03122	0.003656 to 0.1268	0.0435
β2	PSA	0.001975	0.01408	−0.02711 to 0.03022	0.8835
β3	Prostate volume	0.006929	0.007144	−0.007537 to 0.02080	0.3321
β4	Biopsy Gleason score	−1.150	0.2775	−1.735 to −0.6413	<0.0001
β5	Tumor—right lobe (%)	−0.01084	0.01247	−0.03602 to 0.01322	0.3848
β6	Tumor—left lobe (%)	0.02977	0.01219	0.006468 to 0.05483	0.0146

**Table 3:** Logistic regression parameter estimates in the subgroup with PSA > 10 ng/mL.

Parameter Estimates	Variable	Estimate	Standard Error	95% CI (Profile Likelihood)	*p* Value
β0	Intercept	8.453	4.641	−0.09811 to 18.33	
β1	PSA	0.03881	0.02577	−0.006135 to 0.1047	0.1321
β2	Prostate volume	−0.005706	0.01169	−0.02955 to 0.01613	0.6253
β3	Biopsy Gleason score	−1.761	0.6148	−3.122 to −0.7041	0.0042
β4	Tumor right lobe (%)	−0.05712	0.02265	−0.1068 to −0.01651	0.0117
β5	Tumor left lobe (%)	0.03719	0.02037	−0.0002598 to 0.08160	0.0679

**Table 4:** Logistic regression parameter estimates in the subgroup with PSA < 10 ng/mL.

Parameter Estimates	Variable	Estimate	Standard Error	95% CI (Profile Likelihood)	*p* Value
β0	Intercept	1.124	2.678	−4.046 to 6.554	
β1	PSA	−0.1612	0.1456	−0.4575 to 0.1191	0.2681
β2	Prostate volume	0.01341	0.01112	−0.008328 to 0.03639	0.2276
β3	Biopsy Gleason score	−0.8602	0.3097	−1.516 to −0.2897	0.0055
β4	Tumor right lobe (%)	0.02145	0.01849	−0.01482 to 0.05837	0.2459
β5	Tumor left lobe (%)	0.02322	0.01980	−0.01555 to 0.06276	0.2408

## Data Availability

The data used in this study are available upon reasonable request from the authors.

## Abbreviations

The following abbreviations are used in this manuscript:

PCa	Prostate cancer
PSA	Prostate-specific antigen
PSAD	PSA density
MRI	Magnetic resonance imaging
mpMRI	Multiparametric magnetic resonance imaging
TRUS	Transrectal ultrasound
RARP	Robot-assisted radical prostatectomy
RP	Radical prostatectomy
ISUP	International Society of Urological Pathology
DRE	Digital rectal examination
IIEF-5	International Index of Erectile Function-5
cT	Clinical tumor stage
